# Nitric Oxide Detection Using a Chemical Trap Method for Applications in Bacterial Systems †

**DOI:** 10.3390/microorganisms11092210

**Published:** 2023-08-31

**Authors:** Marilene Silva Oliveira, Karina F. D. N. Santos, Railane Monteiro de Paula, Luciana C. Vitorino, Layara A. Bessa, Alexander Greer, Paolo Di Mascio, João C. P. de Souza, Claudia C. G. Martin-Didonet

**Affiliations:** 1 Instituto Federal de Educação, Ciência e Tecnologia Goiano, Departamento de Agroquímica, Campus Rio Verde, Rio Verde 75901-970, GO, Brazil; layara.bessa@ifgoiano.edu.br (L.A.B.); joao.perbone@unesp.br (J.C.P.d.S.); 2 Câmpus Henrique Santillo de Ciências Exatas e Tecnológicas Henrique Santillo, BR 153 n° 3105—Fazenda Barreiro do Meio, Anápolis 75132-903, GO, Brazil; kabiomol@gmail.com (K.F.D.N.S.); railanemonteiroo@gmail.com (R.M.d.P.); ccdidonet@gmail.com (C.C.G.M.-D.); 3 Simple Agro Corporation, rua Parque General Borges Forte, 400, Jardim Goiás, Rio Verde 75903-421, GO, Brazil; 4 Department of Chemistry, Brooklyn College, City University of New York, Brooklyn, NY 11210, USA; agreer@brooklyn.cuny.edu; 5 The Graduate Center, City University of New York, New York, NY 10016, USA; 6 Departamento de Bioquímica, Instituto de Química, Universidade de São Paulo, São Paulo 05508-000, SP, Brazil; pdmascio@iq.usp.br; 7 Departamento de Química, Faculdade de Ciências, Universidade Estadual Paulista, Av. Eng. Luiz Edmundo Carrijo Coube, 14-01, São Paulo 17033-360, SP, Brazil

**Keywords:** nitric oxide, Greiss reaction, fluorescence, chromatography, N_2_-fixing bacteria

## Abstract

Plant growth-promoting bacteria (PGPB) can be incorporated in biofertilizer formulations, which promote plant growth in different ways, such as fixing nitrogen and producing phytohormones and nitric oxide (NO). NO is a free radical involved in the growth and defense responses of plants and bacteria. NO detection is vital for further investigation in different agronomically important bacteria. NO production in the presence of KNO_3_ was evaluated over 1–3 days using eight bacterial strains, quantified by the usual Griess reaction, and monitored by 2,3-diaminonaphthalene (DAN), yielding 2,3-naphthotriazole (NAT), as analyzed by fluorescence spectroscopy, gas chromatography–mass spectrometry, and high-performance liquid chromatography. The Greiss and trapping reaction results showed that *Azospirillum brasilense* (HM053 and FP2), *Rhizobium tropici* (Br322), and *Gluconacetobacter diazotrophicus* (Pal 5) produced the highest NO levels 24 h after inoculation, whereas *Nitrospirillum amazonense* (Y2) and *Herbaspirillum seropedicae* (SmR1) showed no NO production. In contrast to the literature, in NFbHP–NH_4_Cl–lactate culture medium with KNO_3_, NO trapping led to the recovery of a product with a molecular mass ion of 182 Da, namely, 1,2,3,4-naphthotetrazole (NTT), which contained one more nitrogen atom than the usual NAT product with 169 Da. This strategy allows monitoring and tracking NO production in potential biofertilizing bacteria, providing future opportunities to better understand the mechanisms of bacteria–plant interaction and also to manipulate the amount of NO that will sustain the PGPB.

## 1. Introduction

Rhizobacteria that establish positive interactions with roots are referred to as plant growth-promoting bacteria (PGPB) and are promising candidates for building biofertilizers useful for sustainable agriculture practices [1]. PGPB can directly promote plant growth through performing biological nitrogen fixation (BNF), scavenging soil nutrients (phosphate solubilization and/or siderophore production), and producing phytohormones including indole-3-acetic acid (IAA), gibberellin, and cytokinins that induce morphological and physiological changes in the roots. They can also function indirectly by reducing the negative impact of pathogens [2,3].

The benefits of biofertilizers originate from the symbiosis of legumes with bacteria, such as the *Bradyrhizobium* genus [4]. Other bacteria, including *Azospirillum, Gluconacetobacter*, *Azoarcus*, *Enterobacter*, *Herbaspirillum, Burkholderia*, and *Rhizobium*, have been reported as potential biofertilizers [5,6,7,8,9,10]. Rhizobacteria, including *Bradyrhizobium* and *Rhizobium*, are responsible for nitrification–denitrification, where nitric oxide (NO) is a key intermediate. During denitrification, nitrate (NO_3_^−^) is reduced to nitrite (NO_2_^−^), NO, nitrous oxide (N_2_O), and molecular nitrogen (N_2_) through the actions of nitrate reductase (reduces NO_3_^−^ to NO_2_^−^), nitrite reductase (reduces NO_2_^−^ to NO), nitric oxide reductase (reduces NO to N_2_O), and nitrous oxide reductase (reduces N_2_O to N_2_) [11]. Conversely, nitrification involves the oxidation of a nitrogen compound, primarily ammonia (NH_3_), to NO_2_^−^, with hydroxylamine (NH_2_OH) as a key intermediate. Nitrification enzymes include ammonia monooxygenase (converts NH_3_ to NH_2_OH) and hydroxylamine oxidoreductase (converts NH_2_OH to NO_2_^−^), along with nitrite and NO reductases [12]. Studies under aerobiotic and anaerobiotic conditions have shown that diazotrophic bacteria, including the *Azospirillum* genus, can use NO_3_^−^, NO_2_^−^, or nitrous oxide (N_2_O) as final electron acceptors [13]

NO is a reactive gaseous molecule with many important biological functions. It is present in all living organisms and can act as a signaling molecule at low concentrations (nM levels). In plants, NO signaling controls growth, development, and plant defense responses to pathogens. In bacteria, NO also induces a defense response against pathogens and may protect against oxidative stress [14,15]. From a plant–bacteria interaction perspective, NO is produced in nodules where it acts as a signaling molecule, regulates gene expression, or acts as an efficient inhibitor of nitrogenase [16]. NO can alleviate oxidative stress caused by antibiotic processes and BNF due to its signaling action [15,17].

Common methods for NO detection in bacteria include the Griess method, electron paramagnetic resonance (EPR), electrochemical methods, and fluorescent probes. These methods have been used for NO detection in plants inoculated with PGPB [18]. In studies on *Azospirillum brasilense* using fluorescence probes, 4,5-diamino fluorescein diacetate (DAF-2D) has been developed for NO detection, quantification, and mechanism elucidation [19]. Inoculants containing *A. brasilense* show evidence of NO mediation of the indole-3-acetic acid (IAA) signaling pathway to increase lateral and adventitious root formation in tomato plants [19,20].

**Scheme 1:**
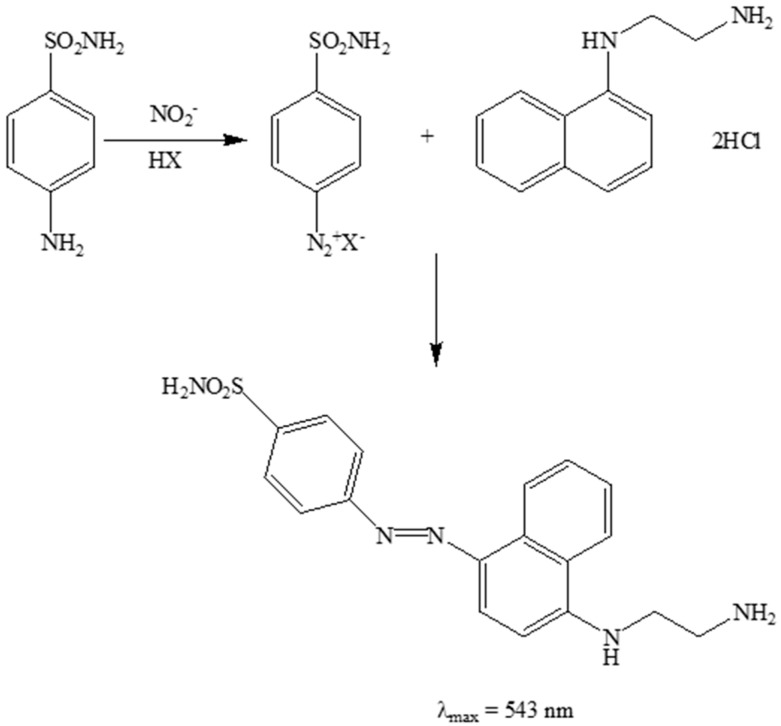
Reaction steps involved in the Greiss method (Nagano, 1999 [21]).

Nitrogen oxide species are produced because NO reacts rapidly with O_2_ and H_2_O, mainly forming NO_3_^−^ and NO_2_^−^. An indirect method for NO determination involves the spectrophotometric measurement of its decomposition products NO_3_^−^ and NO_2_^−^. This method, known as the Greiss reaction, involves the reduction of NO_3_^−^ to NO_2_^−^. NO_2_^−^ subsequently reacts with sulfanilamide in an acidic medium to produce a diazonium ion that reacts with *N*-(1-naphthyl) ethylenediamine to form an azo-chromophore compound that exhibits strong absorbance at 543 nm (Scheme 1) [21]. 

The Greiss method is a simple, indirect colorimetric method for detecting NO in biological systems, requiring only a UV-visible spectrophotometer; however, it is limited to sensitivities of 0.1–1.0 µmol/L [21]. 

To improve NO detection sensitivity, another method was developed based on the diamino aromatic compound 2,3-diaminonaphthalene (DAN) as an indicator of NO formation [22]. DAN is relatively non-fluorescent and reacts rapidly with N_2_O_3_ generated by nitrite under acidic conditions or the interaction of NO with oxygen to produce the strongly fluorescent product 2,3-naphthotriazole (NAT). DAN detection exhibits NO detection limits in the nanomolar range of 10 nM to 10 μM [21,22,23] and can be equal to or 10-fold more sensitive than the Griess method. Therefore, both methods were used as a strategy to improve the determination of NO production in bacteria. 

Reports have shown an improved understanding of NO production in biological systems based on the DAN method. Wada et al. (2002) [24] showed that NO can be detected in *Agave pacifica* plant cells using DAN with high-performance liquid chromatography (HPLC) analysis and fluorescence detection. Furthermore, the DAN method is efficient for NO detection in fungal and bacterial cells, although studies have mainly focused on plant growth-promoting symbionts, including mycorrhizae, nutrient solubilizers, and diazotrophs [25,26,27,28,29]. 

However, few studies have investigated NO detection in diazotrophic biological systems, including bacteria and cyanobacteria [20,30,31]. Thus, it is important to improve the suitable methods for these systems. The rapid detection of NO synthesis in these bacteria is essential to better understand the effects of NO on plant–bacteria interactions and BNF [32]. Therefore, we tested the hypothesis that N_2_-fixing bacteria can synthesize different amounts of NO in the presence of KNO_3_ and at different cultivation times and that NO can be detected by fluorescent probes. Bacteria that form associations with grasses and soybeans were selected; the Greiss reaction and DAN chemical trap were used to quantify and monitor the synthesized NO levels, respectively. Fluorescent probes have provided abundant information regarding the location and mechanism of reactive species produced in biological systems due to their detection sensitivity. 

Herein, an improved DAN chemical trap and the Griess method were applied as facile and inexpensive methods to identify PGPB that produce NO. This is an important advancement because only a few species and strains have been evaluated for NO production to date. The method developed in our study will help identify strains that can be potentially used as biofertilizers for important agricultural plants. In addition, the methods can be applied in future studies to monitor and manipulate NO production on plant–bacteria interactions. 

## 2. Materials and Methods

### 2.1. Bacterial Growth Conditions

Eight strains of bacteria were used: *A. brasilense* (FP2); *A. brasilense* (HM053); *Herbaspirillum seropedicae* (SmR1); *Nitrospirillum amazonense* (Y2); *Rhizobium tropici* (BR322); *Agrobacterium fabrum* (L40); *Gluconacetobacter diazotrophicus* (Pal 5); and *Agrobacterium fabrum* (R5). The bacteria were grown in 96-well plates containing liquid NFbHP–NH_4_Cl–lactate [25] supplemented with 10 mM KNO_3_ at 30 °C with shaking (140 rpm). After 24, 48, and 72 h of growth, the bacteria were collected and centrifuged, and then the supernatant was used for NO measurements.

### 2.2. Determining NO Production in Bacteria via the Greiss Reaction and Fluorescence

Bacterial NO production was quantified using the Griess method [33] and monitored using fluorescence based on the classical reactions involving NO and DAN. 

The Griess method was performed according to the procedures described in the literature [34], and samples were analyzed after 24, 48, and 72 h of growth in liquid media. The observed NO values were standardized for the bacterial growth using the total protein concentration by the Bradford method [35]. These values were expressed as µg mL^−1^ of NO/µg mL^−1^ of total protein.

For fluorescence detection, the bacteria were grown under the same conditions described above, except with the addition of the NO trap DAN. The following incubation conditions were used: (1) NFbHP–NH_4_Cl–lactate medium with 10 mM KNO_3_ and 80 µg mL^−1^ DAN with bacteria; (2) NFbHP–NH_4_Cl–lactate medium with 10 mM KNO_3_ and 80 µg mL^−1^ DAN without bacteria; and (3) NFbHP–NH_4_Cl–lactate medium with 10 mM KNO_3_ without DAN and bacteria. Conditions 2 and 3 were used as controls. After incubation, the bacteria were centrifuged and the supernatant was deposited in a 96-well ELISA plate (suitable for fluorescence) and analyzed using SpectraMax Paradigm Multi-Mode Microplate Reader (Molecular Devices, San Jose, CA, USA) with excitation and emission at 365 and 465 nm, respectively. 

### 2.3. Monitoring NO Production in the NFbHP-NH_4_Cl-Lactate Medium

DAN fluorescence analyses were performed using diethylamine NONOate diethylammonium salt (DEA NONOate), a NO generator. The following conditions were applied: (1) no sample; (2) water and NFbHP–NH_4_Cl–lactate medium; (3) DAN 0.005 mg mL^−1^; (4) DAN 0.005 mg mL^−1^ with DEA NONOate 0.005 mg mL^−1^; (5) DAN 0.005 mg mL^−1^ with DEA NONOate 0.0125 mg mL^−1^; and (6) DAN 0.005 mg mL^−1^ with DEA NONOate 0.025 mg mL^−1^. Conditions 1 and 2 were used as controls. Analyses were performed using a 96-well ELISA plate and SpectraMax Paradigm Multi-Mode Microplate Reader (Molecular Devices) equipment with excitation and emission wavelengths of 365 and 465 nm, respectively.

### 2.4. Monitoring NO Production in Bacteria by Chromatography

This procedure was based on detecting NAT complex formed in bacteria using gas chromatography–mass spectrometry (GC-MS) and Varian HPLC. 

NAT was extracted with toluene by adding 1 mL toluene to a 1 mL bacterial sample. The phases were separated, and the toluene phase was inserted in a GC-MS injection vial.

For GC-MS NAT detection, a Perkin Elmer Claris SQ 8T instrument with an Elite-5 ms Perkin Elmer column (30 m × 0.25 mm I.D. × 0.25 µm) was used. The oven was programmed from an initial temperature of 60 °C rising to 240 °C at a rate of 3 °C min^−1^ and then to 280 °C at a rate of 15 °C min^−1^, with He as the carrier gas. The injector, interface, and source temperatures were 240 °C, 250 °C, and 250 °C, respectively. The injection volume was 1 μL, with a split of 20 mL min^−1^. 

HPLC was used to evaluate the products formed in the DEA NONOate experiment, with method development featuring an acetonitrile/water elution system with 30–90%, with an acetonitrile gradient for the first 10 min, followed by 30–90% acetonitrile for an additional 2 min, and the remaining with 30% acetonitrile until 15 min, at a flux rate of 1 mL/min. A C18 Luna Phenomenex column (250 × 4.6 mm, 5 µm i.d.) was used with an injection volume of 20 µL sample and a standard injection volume. 

### 2.5. Statistical Analysis

NO production by bacterial strains was evaluated in a 10 × 3 factorial scheme, with 8 bacterial strains at 3 time points after inoculation (24, 48, and 72 h). The fluorescence data obtained from NAT production by the different bacterial strains were also evaluated in a 10 × 3 factorial scheme, including the 8 bacterial strains and 2 controls at 3 time points after inoculation (24, 48, and 72 h). Data were analyzed using analysis of variance (ANOVA), and means were compared using Tukey’s test at a 5% probability.

## 3. Results

### 3.1. Greiss and 2,3-Diaminonaphthalene (DAN) Assays

NO is an important messenger in biological systems, and we aimed to determine its production in different bacterial strains using a Greiss assay and a fluorescent probe to quantify and monitor the NO generation processes in the presence of KNO_3_. 

**Figure 1:**
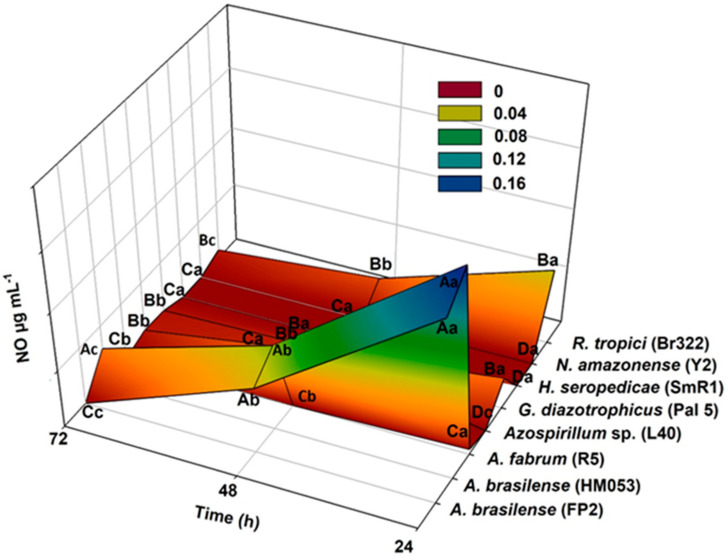
Nitric oxide (NO) quantification for eight strains of bacteria grown for 24, 48, and 72 h by Griess assay. The capital letters indicate the comparison of the bacteria within the same time, and the lower-case letters indicate those between different time points for the same bacterium. Means followed by the same letter do not significantly differ from each other according to Tukey’s test at 5% probability.

**Figure 2:**
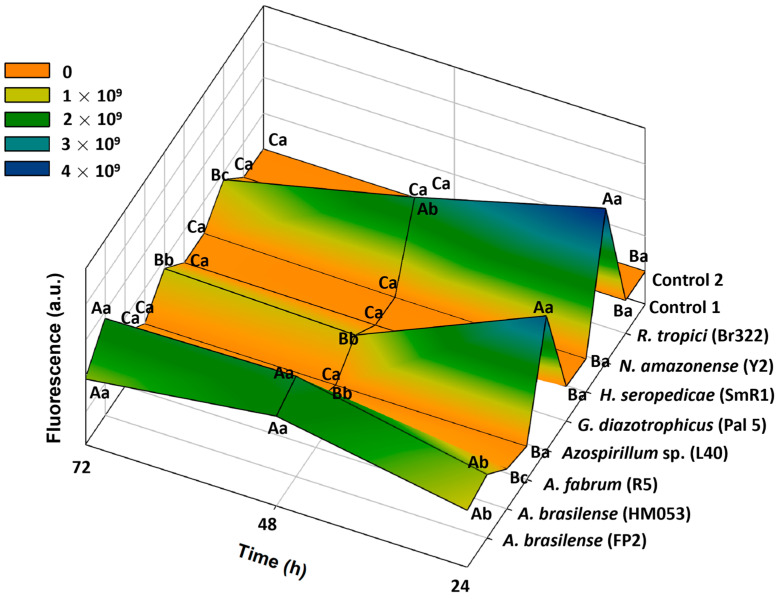
Fluorescence analysis to semi-quantification for 2,3-naphtotriazole (NAT) complexes for eight bacteria strains after 24, 48, and 72 h of incubation. Control 1—Medium supplemented with 10 mM KNO_3_ and 80 µg mL^−1^ of 2,3-diaminonaphthalene (DAN). Control 2—Medium with 10 mM KNO_3_. The capital letters indicate a comparison of bacteria at the same time points, and the lower-case letters indicate the different time points for the same bacteria.

The colorimetric quantification results obtained by the Griess assay corroborated the semi-quantitative fluorescence results. The bacterial strains synthesized different NO amounts depending on incubation time, as shown by the Griess method and fluorescence results (Figure 1 and Figure 2). Furthermore, after 24 h of inoculation, *A. brasilense* HM053 and FP2 synthesized the highest amounts of NO (0.1435 and 0.1259 µg mL^−1^, respectively), followed by *R. tropici* Br 322 (0.0521 µg mL^−1^) and *G. diazotrophicus* Pal 5 (0.0215 µg mL^−1^; Figure 1). The same strains showed the highest NO production in the fluorescent assay at 24 h (Figure 2). During the second evaluation period, the FP2, HM053, Br 322, and Pal 5 strains showed decreased NO production, with FP2 and HM053 still showing the highest NO levels (0.0594 and 0.0458 µg mL^−1^, respectively; Figure 1). At 72 h after inoculation, a significant decrease in NO production was observed for the FP2, Br 322, and Pal 5 strains compared to HM053, which maintained a higher NO production (0.0240 µg mL^−1^; Figure 1). These results were similar to the fluorescence data (Figure 2). *N. amazonense* (Y2) and *H. seropedicae* (SmR1) did not produce NO at any time point, as indicated by the Griess and fluorescent assays (Figure 1 and Figure 2). *A. fabrum* (R5) showed a low (0.0036 µg mL^−1^ NO) production at 24 h of growth and no NO production at other times, similar to the fluorescence results (Figure 1 and Figure 2). 

In summary, NO synthesis decreased with cultivation time for all strains except for the *Azospirillum* sp. (L40) strain, which did not produce NO after 24 h. However, after 48 and 72 h, L40 showed NO concentrations of 0.0239 and 0.0058 µg mL^−1^, respectively (Figure 1); this finding deviated from the fluorescence results, which did not show significant NAT formation at any time point (Figure 2). Thus, the Griess reaction is more efficient for capturing NO than DAN, whereas the DAN method is more sensitive for measuring low NO concentrations (Figure 2), thereby explaining the divergence in the results.

The fluorescence results confirmed fluorescent complex formation from the reaction of NO with DAN to yield the NAT complex. After 24 h of incubation in DAN-containing media, the highest average fluorescence rates were observed for *R. tropici* Br 322 (3.9 × 10^9^ a.u.) and *G. diazotrophicus* Pal 5 (2.9 × 10^9^ a.u.), followed by those of *A. brasilense* FP2 (6.9 × 10^8^ a.u.) and HM053 (8.8 × 10^8^ a.u.; Figure 2). 

A similar pattern was observed in the second evaluation, when the averages for the fluorescence index were 2.0 × 10^9^ a.u. for *R. tropici* Br 322, 1.6 × 10^9^ a.u. for *A. brasilense* FP2, and 1.9 × 10^9^ a.u. for HM053, with a significant increase in fluorescence for *A. brasilense* (Figure 2). After 72 h of incubation, high fluorescence was observed only for *A. brasilense* strains FP2 and HM053 (9.3 × 10^8^ and 1.8 × 10^9^ a.u., respectively; Figure 2). 

*Azospirillum* sp. (L40), *N. amazonense* (Y2), *H. seropedicae* (SmR1), and *A. fabrum* (R5) did not show significant NAT formation over time, whereas *A. fabrum* (R5) showed low fluorescence at 24 h after inoculation (3.7 × 10^8^ and 5.9 × 10^8^ a.u., respectively for 24 and 48 h; Figure 2). 

### 3.2. GC-MS Analyses

**Figure 3:**
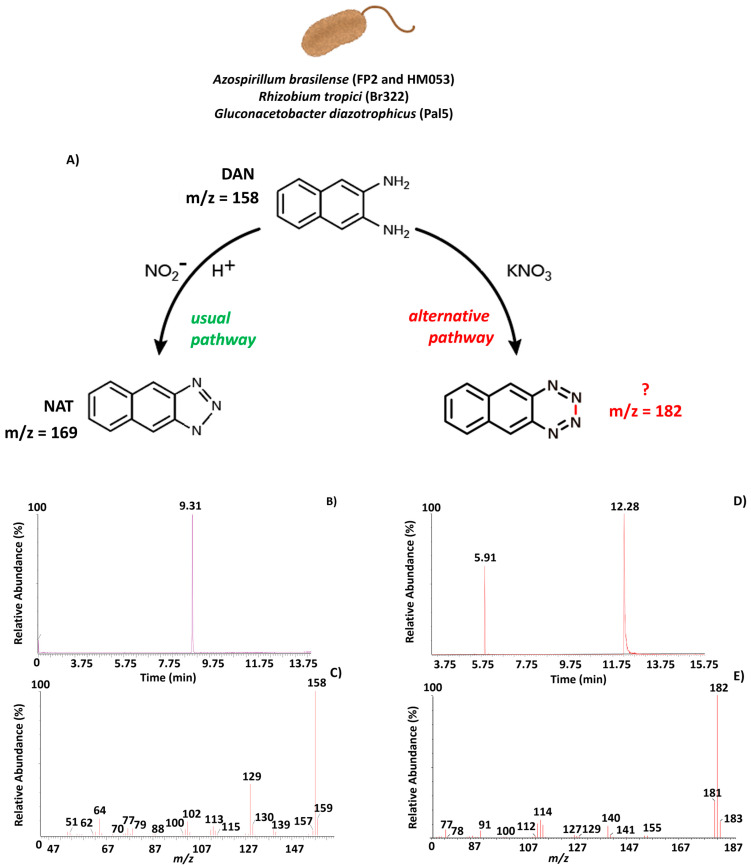
GC-MS analyses for NAT complex detection. (**A**) Typical and alternative metabolic pathways stimulated by supplying KNO_3_ as a nitrogen source for N_2_-fixing bacteria. (**B**,**C**) Total ion chromatogram and mass spectrum obtained for DAN. (**D**,**E**) Total ion chromatogram and mass spectrum obtained by DAN trapping of nitric oxide (NO) produced by bacteria *A. brasilense* (FP2 and HM053), *R. tropici* (Br 322), and *G. diazotrophicus* (Pal5). ? = unknown product.

After different incubation times in the presence of DAN, the samples were analyzed by GC-MS to confirm NAT complex formation. According to the mechanism of NO trapping by DAN, NAT is the dominant product formed; however, another product with additional nitrogen was detected by GC-MS (Figure 3). The same product was observed for *A. brasilense* FP2 and HM053, *R. tropici* Br 322, and *G. diazotrophicus* Pal5, which showed fluorescence after 24 and 48 h of growth (Figure 3D,E). Therefore, an alternative metabolic pathway stimulated by supplying KNO_3_ as a nitrogen source for N_2_-fixing bacteria can be proposed. In this pathway, DAN is converted into a compound with a molecular mass ion of 182 Da, namely, 1,2,3,4-naphthotetrazole (NTT) instead of NAT, given the presence of reactive nitrogen species (RNS) in the medium (Figure 3A,D,E).

In the total ion chromatogram of DAN, a peak was observed at 9.31 min, and the mass spectrum showed the molecular mass ion of DAN, *m*/*z* = 158 Da (Figure 3B,C). After extraction of the bacterial supernatant with toluene, the peak at 12.28 min originated from the product formed in the reaction of DAN with NO (Figure 3D). The ion *m*/*z* = 182 Da corresponds to the molecular mass ion of the product; however, to obtain *m*/*z* = 182 Da, two additional nitrogen atoms in the DAN structure would be necessary to form NTT. In addition, the mass spectrum shows the loss of two nitrogen atoms with *m*/*z* = 155 Da, of a third nitrogen with *m*/*z* = 127 Da, and, lastly, of another nitrogen with *m*/*z* = 140 Da as fragment ions (Figure 3E). The other peak at 5.91 min corresponds to impurities present in the toluene solvent (Figure 3D).

### 3.3. Validation Method

**Figure 4:**
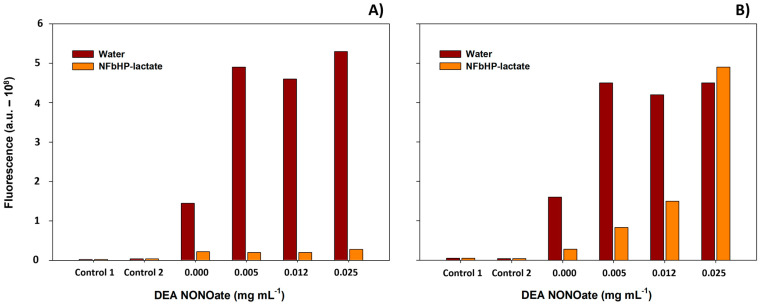
Fluorescence analyses for nitric oxide (NO) production by Diethylamine NONOate diethylammonium salt (DEA NONOate) generator using DAN as a probe in water and NFbHP–NH_4_Cl–lactate. (**A**) Data after 30 min of incubation. (**B**) Data after 24 h of incubation. Wavelength excitation and emission at 365 and 465 nm. Control 1—No sample. Control 2—Water and NFbHP–NH_4_Cl–lactate.

**Figure 5:**
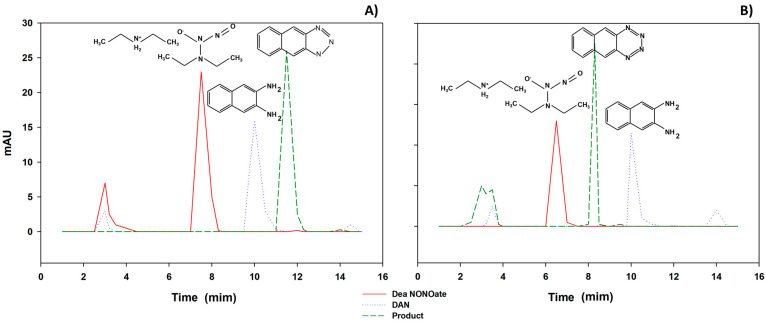
HPLC chromatogram obtained from the reaction of 0.025 mg mL^−1^ DEA NONOate with 0.005 mg mL^−1^ DAN. (**A**) In the acid medium after 30 min incubation. (**B**) In NFbHP–NH_4_Cl–lactate medium after 24 h incubation.

The use of DAN as a bacterial probe was validated using DEA NONOate in water and NFbHP–NH_4_Cl–lactate media. DEA NONOate was used to monitor NO production in the culture medium. This compound can also produce NO in 0.1 mol L^−1^ phosphate buffer pH 7.4 (*t*_1/2_ = 16 min at 22–25 °C, *t*_1/2_ = 2–4 min at 37 °C), where its decomposition is nearly instantaneous at pH 5.0. Fluorescence analysis was performed with excitation and emission at 365 and 465 nm, respectively, showing product formation in water and culture medium (Figure 4 and Figure 5).

The fluorescence intensity increased with increasing NO generator concentrations in stirred water for 30 min. In the culture medium, the fluorescence intensity remained unchanged except after 24 h of stirring, at which time increased fluorescence was observed due to complex formation. The observed fluorescence increased as a function of NO generator concentration (Figure 4A,B). This validation test provided evidence for incubation in NFbHP–NH_4_Cl–lactate medium as a method for detecting NO in bacterial systems because no NO release by the medium was observed in the absence of the generator. 

Thus, NO release occurs when bacteria are stressed in the presence of KNO_3_, as shown by DAN analysis in the medium without NO generator, such as DEA NONOate or KNO_3_, which showed no product formation. The product was observed when the DEA NONOate concentration was increased.

In the sample with only 0.005 mg mL^−1^ DAN, an intense fluorescence was observed due to the compound exhibiting fluorescence in the wavelength used to obtain the results with excitation and emission at 365 and 465 nm, respectively. In the culture medium, the complexity of components quenches the DAN fluorescence.

The samples in the NFbHP–NH_4_Cl–lactate medium used in the fluorescence experiment were also analyzed by HPLC, confirming that a different product was formed during NO trapping (Figure 5B). As shown in the chromatogram in Figure 5, the NAT complex formed in the acid medium has a retention time of 11.8 min (Figure 5A), while the complex obtained in the culture medium has a retention time of 8.1 min (Figure 5B). These results also showed that all DEA NONOate was consumed and all DAN reacted with NO in the system since the DAN presented the retention time of 10.1 min initially (Figure 3A,B,E) and disappeared completely after 30 min and 24 h of reaction (Figure 3A,B,E). Thus, the samples were also analyzed by GC-MS, and it was confirmed that the same product with *m*/*z* 182 Da was formed (Figure 3 and Figure 5). Considering a 1:2 stoichiometric reaction ratio (DAN:NO), a concentration of 0.025 mg mL^−1^ NO was determined (Figure 5) to react completely with DAN, showing that two nitrogens are added to the DAN molecule, in contrast to the NAT complex that is typically formed.

## 4. Discussion

The Griess assay, EPR, fluorescent probes, and electrochemical sensors are established NO detection methods in bacteria [18]. In addition, fluorescent probes can be used to monitor the real-time NO production in the root tissues of inoculated plants by microscope analyses. NO production by *Mesorhizobium loti* in *Lotus japonicus* and *Medicago sativa* roots has been suggested as a specific recognition signal between plants and bacteria [36,37]. In *M. truncatula*–*Sinorhizobium meliloti* symbiosis, NO production was detected by confocal microscopy using a DAF probe at different root sites and in the nodule fixation zone during the infection process [38,39]. For *Azospirillum* inoculated in tomato, a DAF probe was also used for NO detection, confirming that NO accumulation mainly occurred in vascular tissues and subepidermal root cells [20]. 

NO synthesis in bacteria is a mechanism associated with denitrification, which is an important part of the nitrogen cycle wherein bacteria reduce NO_3_^−^ to N_2_O and N_2_ to obtain energy or achieve redox balance during anaerobic respiration [40]. 

The DAN assay has already been demonstrated as a plausible model for NO production monitoring in *Escherichia coli* [41,42]; however, it has not been previously tested in rhizobacteria. *E. coli* RF1005 grown in Luria–Bertani (LB) medium supplemented with 12 mM KNO_3_ showed initial rates of NO production of 10 nmol min^−1^ per mg of cell protein and a maximum concentration of 200–300 µmol L^−1^ [41,42]. 

In this study, the main goal was to show that the Griess and DAN assays were efficient for NO detection; here, we confirmed the ability of *A. brasilense* HM053 and FP2, *R. tropici* Br322, and *G. diazotrophicus* Pal5 to synthesize NO in NFbHP–NH_4_Cl–lactate medium supplemented with 10 mM KNO_3_. This study is the first to establish DAN fluorescent probes as an inexpensive and reliable NO detection method that can be applied in different rhizobacteria species and in studies that aim to understand the mechanisms associated with plant–bacteria interactions. 

Denitrification can also occur under fully aerobic conditions [43], as shown for *A. brasilense* Sp245, which can produce NO by aerobic denitrification in an OAB medium containing NO_3_. The NO concentration was approximately 25-fold higher in NO_3_^−^-containing media (120 nmol NO per gram of bacteria) than in OAB with NH_4_^+^ (4.2 nmol NO per gram of bacteria) as detected by EPR spectroscopy [19]. This suggests that NO_3_^−^ is the main source of NO production by *A. brasilense*. In our work, NFbHP–NH_4_Cl medium supplemented with KNO_3_, an extra N source, was used to grow the bacteria strains; after 24 h to 72 h, NO quantification was performed using the Griess assay. NO concentrations ranged from 0.1435 for *A. brasilense* HM053 to 0.0036 for *A. fabrum* R5, normalized to total protein content.

Moreover, genes associated with denitrifying activity have been identified in *A. brasilense* plasmids (the *nap*, *nir*, *nor*, and *nos* genes) [44,45]. Moreover, in these bacteria, multidomain metalloprotein NO synthase might be present, producing NO aerobically by oxidizing arginine. Studies on these bacterial NO synthases revealed new roles for NO as a participant in toxin biosynthesis, protecting against oxidative stress, and in radiation damage recovery regulation [46].

Important functions have been attributed to NO in PGPB. Molina-Faveiro et al. (2008) [19] and Creus et al. (2005) [20] observed NO production in *A. brasilense* and concluded that the bacterially-derived NO is involved in the induction of lateral roots in tomato plants. 

NO produced by *A. brasilense* is potentially involved in biofilm formation, which is important for root colonization and growth; moreover, considerable quantities of endogenous NO can be formed by *A. brasilense* by different metabolism using two N sources, such as NH_4_Cl and KNO_3_. Thus, a correlation between the production of NO_3_^−^ and the two N sources used was concluded [47]. However, studies have suggested that changes in root architecture induced by *A. brasilense* are associated with NO-promoted activation of IAA signaling pathways [48,49,50]. Previous studies have demonstrated a cross-talk between IAA and NO in the rhizosphere, where IAA-producing and NO-producing bacteria such as *A. brasilense*, *R. tropici*, and *G. diazotrophicus* may participate [51,52]. These species may have genes/proteins that are responsive to NO stimulation, which directly impacts the quorum sensing system and biofilm formation [53], which are processes key to plant–bacteria interaction [54].

For the *Rhizobium* genus, NO production occurs during the symbiosis process between *Rhizobium* and legumes, from the initial interaction to nitrogen fixation nodule formation [55]. Signorelli et al. (2020) [17] studied the role of NO in legume–rhizobia symbiosis and inferred that although this reactive species can reduce nitrogenase activity, NO exerted positive effects on BNF. Notably, the negative effects of NO require direct interaction with nitrogenase, whereas the positive effects are related to signaling functions, which can amplify beneficial processes. Thus, the detection of NO synthesis by nitrogen-fixing bacteria can be used to detect the ability of the bacteria to amplify BNF, which would be particularly interesting for strain choice in agriculture. 

In contrast to NO-producing bacteria such as *A. brasilense*, the *H. seropedicae* (SmR1) genome was completely sequenced, and no genes encoding denitrification enzymes, such as nitrous oxide reductase, [56] were identified. Baldani et al. (1986) [6] also found no evidence of denitrification in *H. seropedicae*. 

Regarding *N. amazonense*, Kloos et al. (2001) [57] evaluated the Y1 strain and found no active genes for denitrification, a finding consistent with the results reported herein. 

In general, the synthesis of RNS in NO-producing bacteria decreased or did not occur after 24 h of incubation, as indicated by the detection of the reaction product with DAN. Moreover, NO synthesis as a signaling molecule appears to be a rapid transient process, as it is rapidly converted into important biological derivatives, including nitrogen dioxide (NO_2_), NO_3_^−^, NO_2_^−^, and others [58]. NO signaling can activate metabolic stress response pathways, including those that protect from oxidative stress and convert NO into metabolic molecules by detoxification, DNA deamination, thiol S-nitrosation, and Fe-S nitrosylation [59]. Thus, when N_2_-fixing bacteria were cultivated in NFbHP–NH_4_Cl–lactate medium supplemented with KNO_3_ under aerobic conditions, transient NO formation occurs during denitrification through the action of nitrite reductases [60].

Herein, the NO produced in the presence of KNO_3_ in the bacterial growth in NFbHP–NH_4_Cl–lactate medium was studied, wherein a reaction with DAN formed a fluorescent compound with a molecular ion of 182 Da, NTT. The NO formed by bacteria replaced with that obtained from DEA NONOate after 24 h was shown to be efficient for detecting increases in NO concentration. This mechanism underpins an important tool for monitoring NO production in bacterial culture supplemented with KNO_3_.

The *N*-nitrosation of DAN, which occurs through the action of strong agents such as N_2_O_3_ and N_2_O_4_ produced from NO, can form a highly fluorescent compound, NAT, that offers specific, sensitive, and versatile detection [61]. Ji and Hollocher (1988) [41,42] demonstrated that *E. coli* can catalyze the nitrosation of DAN by NO_2_^−^ in the required presence of NO. Thus, KNO_3_ possibly acts as the substrate for the nitrosating agent formation, which directly promotes DAN nitrosation in the presence of NO. By contrast, Brew and Forsythe (1990) [62] demonstrated that the rate of bacterial nitrosation observed for *Neisseria subflava* was optimal with glucose as an electron donor for NO_2_^−^ reduction to a nitrosating species. Herein, NFbHP–NH_4_Cl–lactate medium was used with lactate as the carbon source. Thus, lactate decarboxylation can provide the electrons necessary for the reduction of nitrogen by-products from the KNO_3_ and NH_4_Cl reactions.

Through bacterial metabolism and the generation of NO via DEA NONOate in NFbHP–NH_4_Cl–lactate medium and the same medium with KNO_3_, DAN was converted into a product with a molecular mass of 182 Da, NTT. Future work will focus on the structural elucidation of this product to help further understand the mechanism by which DAN acts as a NO probe in bacterial systems and its application for monitoring processes related to KNO_3_ metabolism by N_2_-fixing bacteria.

In this study, we present results that show the production of NO metabolized by bacteria treated with a source of KNO_3_ by indirect detection (Greiss reaction and DAN chemical trap). Notably, in the absence of KNO_3_, NO was not detected.

Our study helps elucidate the NO production mechanism in bacteria because this molecule is an essential component in the agribusiness sector, given its role in promoting plant growth through the BNF mechanism. However, more refined future experiments are needed, such as isotopic labeling and elucidation of the product formed in chemical trapping.

## 5. Conclusions

The results presented herein are consistent with our hypothesis that *A. brasilense* HM053 and FP2, *R. tropici* Br322, and *G. diazotrophicus* Pal5 can produce detectable NO concentrations in NFbHP–NH_4_Cl–lactate medium containing KNO_3_. After 24 h of growth, NO concentrations ranged from 0.1435 µg mL^−1^ (*A. brasilense* HM053) to 0.0036 µg mL^−1^ (*A. fabrum* R5), as determined using the Griess assay. After 48 and 72 h of growth, these strains showed decreased or no production of NO, similar to R5. *Azospirillum* sp. L40 differed from the other strains, not producing detectable amounts of NO at 24 h of growth, but started forming NO after 48 and 72 h. Overall, the Griess and DAN results are mutually consistent, confirming the applicability and reliability of DAN for NO detection. 

The ability to synthesize NO is linked to oxidative stress resistance, which can amplify BNF and promote plant growth. DAN probing of NO is an efficient method for detecting NO production in bacterial systems. Moreover, using NFbHP–NH_4_Cl–lactate as a culture medium added with KNO_3_ results in the recovery of a product different from NAT (naturally produced by the reaction of DAN and NO) with or without bacterial metabolism. This product (1,2,3,4-naphthotetrazole (NTT)) has a molecular mass of 182 Da and may assist in the understanding of mechanisms associated with the action of DAN and in the elucidation of KNO_3_ metabolic pathways as a nitrogen source for NO generation in diazotrophic bacteria. The approaches used herein may contribute to further understanding of processes associated with NO production by bacteria and be applied in future projects to better understand the role of NO in cross-talk between plants and PGPB. 

## Data Availability

Not applicable.
